# 

**Published:** 1887-09-24

**Authors:** 


					PRICE ONE PENNY. T) ^ " ft
No. 52, Vol. II.
September 24th, 1887.
It *
'"Kistered at tlie General Post Office
as a Newspaper.]
IX
Per Annum, 6s. 6d.,post tree.
Monthly Parts, 6d.; post tree, 7$d.
Ditto uer Ann.. 7s. 6d. Dost tree.
2ht finstitution, jfamilg, antr $arocf)ial journal of
l^ogpttalg, asylums, fr all agencies for tf)e flare of tfle gitclt, ?rittctsm & fletos.

				

